# Sustainability of medical assistance in dying provision: Provider perspectives

**DOI:** 10.1017/S1478951526102120

**Published:** 2026-04-13

**Authors:** Cory Byrne, Doruntina Yakoub, Robert Sibbald, Dawn Papanayotou, Marat Slessarev, John Basmaji, Carter Winberg, Ian Ball

**Affiliations:** 1 Schulich School of Medicine, Western University, London, ON, Canada; 2 London Health Sciences Centre, Department of Ethics, London, ON, Canada; 3 Department of Family Medicine, Western University, London, ON, Canada; 4 Department of Medicine, Western University, London, ON, Canada; 5 Department of Epidemiology and Biostatistics, Western University, London, ON, Canada; 6 Office of Academic Military Medicine, Western University, London, ON, Canada

**Keywords:** Medical assistance in dying (MAID), sustainability, resilience, end-of-life care, provider wellbeing

## Abstract

**Objectives:**

Medical assistance in dying (MAID) is a rapidly growing and evolving field. The provision of MAID in Canada has substantially outpaced the number of new providers. While challenges of provision have been well described, little is known about the sustainability of providing this care long term. To fill this gap, we aimed to determine if providing MAID is sustainable while identifying factors that impact provider wellbeing.

**Methods:**

We developed a 20-item Likert scale-based questionnaire that focused on themes of sustainability. We performed descriptive analyses for each question and used Fisher’s exact and Kruskal–Wallis tests to assess differences across provider characteristics. The questionnaire was distributed via a network of MAID navigators and providers in Ontario, Canada.

**Results:**

In total, 38 responses were received from well-experienced clinicians in a variety of specialties. A total of 74% of respondents felt their MAID work was sustainable for the long term. Practitioners strongly enjoyed the work and reported little emotional toll and burnout. While some providers felt the compensation and training were sufficient, others felt it could be improved. Nearly all respondents had someone ethically and clinically knowledgeable about MAID they could go to for support.

**Significance of results:**

Our questionnaire has shown clinicians who are well-experienced and connected to supports report very positive experiences providing MAID and view the work as sustainable. While existing literature and media often emphasize the challenges of MAID, the perspectives of providers highlight a positive experience.

## Introduction

Having been legalized in 2016, medical assistance in dying (MAID) remains a novel practice in Canada. Since its legalization, the demand for MAID has outpaced the number of new providers available. From 2019 to 2023, annual MAID provisions have increased from 5,631 to 15,343 while the number of MAID providers has increased from 1,271 to 2,200 (Canada 2024). In other words, MAID provisions have grown 2.36 times faster than the number of new MAID providers. Additionally, a small proportion of providers complete the majority of cases. Just 16.5% of providers complete 66.4% of Track 1 cases and 58.4% of Track 2 cases (Canada 2024).

In addition to this increasing workload, research to date has focused on the challenges associated with providing MAID (Fujioka et al. 2018). Studies have identified long hours outside conventional workdays, stigma from healthcare and wider communities, limited training, inadequate compensation, and unclear guidelines as major challenges to MAID provision (Beuthin et al. 2018, 2020; Fujioka et al. 2018; Khoshnood et al. 2018; Shaw et al. 2018; Crumley et al. 2021; Mathews et al. 2021; Oczkowski et al. 2021; Stewart et al. 2021; Winters et al. 2021, 2022, 2025; Dholakia et al. 2022). Nevertheless, providers still feel the work is meaningful and emotionally rewarding through upholding patient-centered care and relieving suffering (Beuthin et al. 2018, 2020; Shaw et al. 2018; Mathews et al. 2021; Oczkowski et al. 2021; Stewart et al. 2021; Winters et al. 2021, 2025; Dholakia et al. 2022). However, limited data exist on the long-term sustainability of providing MAID. One survey of the Canadian Association of MAID Assessors and Providers found 20% of respondents consider stopping their work with MAID (Stewart et al. 2021). Concerns of MAID sustainability are prevalent in the literature (Beuthin et al. 2018; Khoshnood et al. 2018).

The sustainability of providing MAID is essential to the accessibility and autonomy of end-of-life care for patients. Continued access to MAID depends on understanding and supporting those who provide it. Listening to provider experiences can correct misconceptions and inform system improvements. Without addressing the factors that affect sustainability, the system may fall short of meeting future patient needs.

## Methods

### Survey

We completed a cross-sectional survey of MAID providers (physicians and nurse practitioners) across Ontario. We generated themes and questions based on the challenges of providing MAID found in the current literature, as well as topics that can affect sustainability. Themes included distress relating to work, training and compensation, as well as workload. The survey collected demographic information on medical specialty, clinical and MAID experience, and time spent on MAID. The questionnaire included 20 questions that were answered with a 5-point Likert scale of “Strongly disagree,” “Disagree,” “Neutral,” “Agree,” and “Strongly agree.” Following established survey methodology, the team pre-tested and pilot-tested the questionnaire and conducted clinical sensibility testing with MAID providers, coordinators, and ethicists (Burns et al. 2008). We disseminated the questionnaire via e-mail to a network of MAID navigators and providers in Ontario. The study used snowball sampling by encouraging participants to share the questionnaire with other MAID providers and networks. We focused on this recruitment method as we felt MAID providers were more likely to complete the survey if the email came from a colleague within MAID. The questionnaire was kept short and a reminder email was sent out to increase response rate (Nakash et al. 2006). The questionnaire was available online via REDCap. Responses were collected from June to August 2024.

### Data analysis

Due to the ordinal nature of the data, we used descriptive analysis and non-parametric analysis as we could not assume equal spacing between Likert responses for parametric analysis. We completed frequency distributions of the responses. Participants could skip questions; the analysis excluded unanswered items for the specific question under consideration. Statistical analyses evaluated differences in provider responses across collected characteristics to identify factors that may influence sustainability. Fisher’s exact test compared responses by provider specialty and case type (Track 1: naturally foreseeable death; Track 2: no naturally foreseeable death). The Kruskal–Wallis test assessed differences in responses by years in healthcare, years as a MAID provider, monthly MAID workload, and number of MAID cases completed in the prior 12 months. All tests were 2-tailed, with statistical significance defined as *p* ≤ 0.05. Analyses used SPSS and R (version 4.4.0). Due to the low number of responses from providers in internal medicine, critical care, emergency medicine, and anesthesiology, these subspecialties were grouped together for the purposes of statistical analysis.

## Results

### Demographics

Thirty-eight MAID providers responded to the questionnaire. Typical respondents were healthcare providers for over 20 years and had been providing MAID for 5–8 years. Table 1 summarizes the respondents’ demographic information.

**Table 1.** Demographic information of respondents

Specialty (%)	
Family medicine	18 (47%)
Palliative medicine	9 (24%)
Nurse practitioner	5 (13%)
Internal medicine	3 (8%)
Critical care and emergency medicine	2 (5%)
Anesthesiology	1 (3%)
**Experience (25th–75th percentile)**	
Median years as healthcare provider	22 (14.5–30)
Median years as MAID provider	7 (5–8)
Median MAID administrations in last 12 months	12 (6.4–28.8)
Median hours spent on MAID per month	15 (5.5–32.5)
**Aspects of MAID involvement (%)**	
Referring patients	15 (39%)
Assessing patients	38 (100%)
Providing counselling/support to the family after MAID	14 (37%)
Track 1 cases	38 (100%)
Track 2 cases	24 (63%)

**Figure 1.**
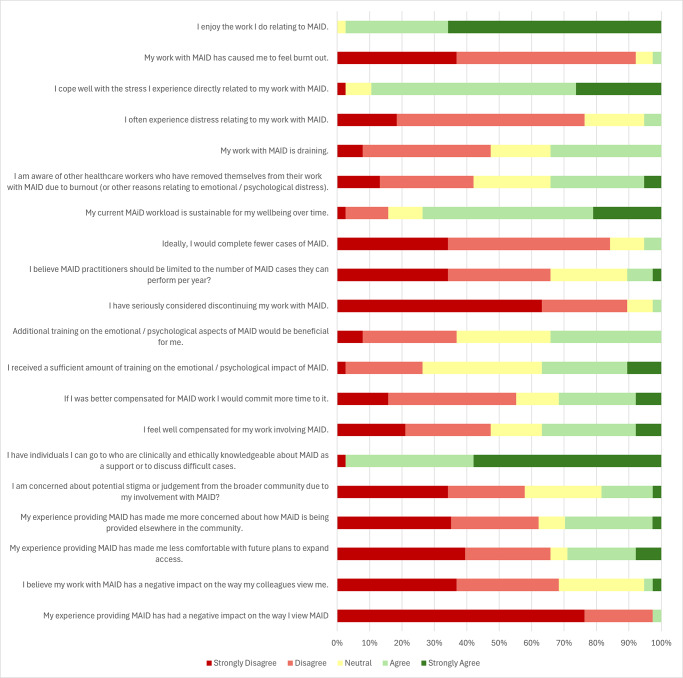
Questionnaire responses of MAID providers (dark red = strongly disagree, light red = disagree, yellow = neutral, light green = agree, dark green = strongly agree).

A visual representation of the results of the survey is demonstrated in Figure 1. Tables 2–23 show statistical findings (supplemental materials). Pertinent findings are described below.

### Wellbeing

Nearly all participants (97%) reported high level of satisfaction relating to their MAID work. Providers who completed Track 2 cases reported greater satisfaction from providing MAID. Likewise, those who enjoyed the work more were more likely to have completed more hours of MAID work each month. Despite some respondents (34%) reporting MAID work as draining, the majority (89%) coped well with the stress of work; only 1 (3%) participant reported having ever been burnt out due to MAID work and 2 (5%) reported experiencing frequent distress related to MAID work. When compared to other specialties, family doctors reported the least amount of burnout. Nearly all respondents (97%) were connected to individuals who are clinically and ethically knowledgeable about MAID for support or to discuss difficult cases. Most respondents (68%) did not feel as though working in MAID negatively impacted the way colleagues viewed them, nor were many (18%) concerned about stigma from the broader community.

### Sustainability

The majority (74%) of providers felt their current MAID workload was sustainable for their wellbeing over time. While 34% were aware of colleagues who had ceased MAID work due to burnout, only 3% had seriously considered withdrawing from MAID practice. Family doctors reported the least intent to discontinue their MAID work, while nurse practitioners felt the work was less sustainable compared to other specialists. Only 5% wished to perform fewer MAID cases, and 11% supported an annual limit on the number of cases a provider can complete. Most respondents did not have concerns over how MAID was being provided elsewhere (62%) or about the future expansion of MAID (66%). Overall, providers’ experience with MAID did not negatively impact their view on MAID (97%). Respondents who provided Track 2 cases were more comfortable with MAID expansion plans compared to those who only completed Track 1 cases.

### Compensation and training

Respondents expressed mixed views regarding compensation for MAID work. While 37% felt well compensated, 47% did not. A portion (32%) would commit more time to MAID if they were better compensated. Those who felt they would spend more time on MAID if they were better compensated were significantly more likely to be healthcare providers with fewer years of experience.

Survey responses similarly indicated mixed views on training. While 37% felt they had received sufficient training on the emotional and psychological impact of MAID, 26% did not. Roughly one third (34%) felt additional training would be beneficial for themselves.

## Discussion

Based on the results of our survey, sustainability in MAID provision appears to be strong. It is important to highlight that this general sense of sustainability was endorsed by experienced, late-career providers who are comfortable in their practice, and connected to MAID navigators. This generates inherent limitations in study results as the views of practitioners who have an abundance of clinical acumen and are well supported in their MAID provisioning will have more positive outlooks on the sustainability of their work. Additionally, our use of snowball sampling may have self-selected people who are similar in attributes. Regardless, the findings of our survey challenge commonly held assumptions that health professionals who provide end-of-life care experience emotional distress or stigma in relation to their work. These misconceptions may stem from a greater stigma and negative discourse surrounding MAID since its introduction. Existing literature highlights that although providers find their work to be rewarding and fulfilling, they still report facing subtle challenges related to legal complexities, institutional barriers, and strained relations with colleagues (Beuthin et al. 2020; Mathews et al. 2021; Close et al. 2023b). These studies highlight the persistent negative attitudes surrounding MAID which can create false narratives of unsustainability within the field of work. Our survey findings help to deconstruct this misconception by demonstrating that while negative stigma and challenges may exist, there are strong positive perceptions amongst the professionals providing MAID. Similar studies to ours have shown MAID providers view the benefits as more substantial than the challenges associated with the work (Beuthin et al. 2020; Stewart et al. 2021; Winters et al. 2025).

An overwhelming majority of respondents expressed no concerns regarding MAID provisions in their communities or the future expansion of MAID services. However, members of the medical and legal communities have historically expressed concern regarding expansion of services citing a lack of clear guidelines, ambiguity in language used in MAID legislation, and the inadvertent targeting of vulnerable individuals (Grant and Downie 2018; Close et al. 2023a). The findings of our survey suggest that MAID providers’ conception of provision and practice have evolved in recent years. With guidelines continuously being refined, training being implemented early on, and MAID providers becoming more comfortable navigating provisions, it seems that expansion is starting to be seen in a more positive light which ultimately works to further strengthen sustainability and longevity of MAID services. It is imperative that future qualitative studies build off these findings to gain a deeper understanding of the factors that are influencing perspectives on current practice and future expansion of MAID. Gaining insight into these attitudes will help future parties in their efforts to bolster sustainability of MAID services.

Stigma surrounding MAID and the prevailing attitudes of both the general and medical community toward the future expansion of MAID services were seen as key factors in optimizing sustainability. The stigma associated with MAID and its administration can be targeted in a multitude of ways. It is important to recognize that this stigma stems not only from interaction with the medical community but also from lived experiences, cultural and religious differences, and personal beliefs. As such, targeting this stigma will require the implementation of multidisciplinary programs. Suggestions from our team include the establishment of training modules for not only physicians but also allied health professionals, public service campaigns highlighting personal patient experiences with MAID, and media training for those reporting on MAID related current events. The development of standardized guidelines and protocols tailored to scope of practice of different health professionals will be vital to facilitating the safe and effective future expansion of MAID services.

Reports published by the Government of Canada indicate that the number of MAID provisions has been steadily increasing since legalization in 2016. These data also highlight that a small proportion of practitioners performed a significant number of MAID procedures. In 2023 alone, only 89 practitioners were responsible for 35.1% of all Track 1 and 28.6% of all Track 2 cases (Canada 2024). This reliance on a small group of providers may further limit the sustainability of MAID availability. Despite the overwhelming proportion of providers in our study endorsing positive perspectives of their work, the growth of MAID requests continues to outpace the increase in providers. Based on our survey findings, lack of financial recognition and structured MAID training may be factors contributing to this discrepancy.

With nearly half of providers reporting being inadequately compensated for their MAID related work, a lack of appropriate compensation may be significantly impacting sustainability of the service and discouraging more healthcare professionals from participating. The literature indicates that Canadian MAID providers view remuneration as an ongoing issue and report discrepancies between time required to provide quality services and compensation (Khoshnood et al. 2018). Lack of financial recognition threatens to discourage providers from contributing to MAID services and jeopardize long-term sustainability of MAID programs.

Roughly one third of survey participants indicated that they would benefit from additional MAID related training. Researchers and educators have called for integrating early MAID education into curricula for future healthcare providers nationwide (Spicer et al. 2017; MacDonald et al. 2018; Shapiro et al. 2024). It has yet to be established whether educational gaps identified in this survey and recent calls for early education stem from a lack formal education, insufficient curriculum implementation, or discrepancies between what is being taught and the realities of clinical practice. Regardless, there is a perceived gap in education identified through the results of this survey and review of relevant literature. Initial and continued education surrounding MAID is essential in breaking down barriers in long-term access to high-quality and sustainable end-of-life care.

With compensation and training being identified as 2 factors that threaten to compromise the sustainability of MAID services, we recommend re-evaluation of remuneration and provincial budgetary allowances for MAID. Similarly, a review of current MAID curriculums in early and continued medical education will be required to better understand the current state of MAID training and guide future recommendations.

### Limitations

One limitation of our study is the small sample size, as we sampled 38 of the 472 unique providers across Ontario (Canada 2024). Likewise, nearly all participants reported strong connections to individuals clinically and ethically knowledgeable about MAID. This likely stems from our recruitment method using a network of MAID navigators to disseminate questionnaires. In this sense, our study demonstrates how individuals who are well connected to supportive resources find the work to be sustainable. This may not be true of all providers, which may limit the generalizability of these findings.

### Future research

Future research should focus on what barriers are preventing additional healthcare providers from participating in MAID provision. They should continue to examine whether a lack of adequate compensation and training are contributing to the growing gap between provisions and new providers. Understanding the nuances related to contextual and personal factors that influence attitudes toward compensation and formal education will be important to guide development of interventions and recommendations. Furthermore, a broader study could better assess additional risk factors and protective factors that may affect sustainability, such as practice location or connectedness to MAID resources and supports. Such research is essential to ensure MAID remains accessible to patients as provisions continue to outpace new providers (Canada 2024).

## Conclusion

This study highlights the perspectives of a small and experienced group of MAID providers regarding sustainability within their realm of practice. Respondents generally reported that they find their MAID related work to be sustainable. Our cohort did not struggle with many of the challenges reported in the literature such as emotional distress, increasing workload, or stigma from colleagues or the community. Results of this study may be explained by the fact that our cohort was not only clinically experienced but also well connected to MAID navigators who are able to support them in their endeavors. This survey identified financial compensation and training as potential areas to improve MAID sustainability. Further research on protective and risk factors that affect sustainability can allow improvements in MAID policies and legislation globally. Ensuring the sustainability of MAID practice is crucial for preserving patient autonomy and access to end-of-life care.

## Supporting information

10.1017/S1478951526102120.sm001 Byrne et al. supplementary material Byrne et al. supplementary material

## Data Availability

The authors confirm that the data supporting the findings of this study are available within the article and its supplementary materials.

## Supplementary material

The supplementary material for this article can be found at https://doi.org/10.1017/S1478951526102120.
