# Supplementary material for: Sustainability of medical assistance in dying provision: Provider perspectives
Source: Palliat Support Care. 2026 Apr 13;24:e105. doi: 10.1017/S1478951526102120 (PMC13166695; doi:10.1017/S1478951526102120)
Supplement: Byrne et al. supplementary material [file S1478951526102120sup001.docx]

|  | **Anesthesia /Critical Care /Internal Medicine** | **Family Medicine** | **Nurse Practitioner** | **Palliative Medicine** | **Total** | **P-value** |
| --- | --- | --- | --- | --- | --- | --- |
|  | **(N=6)** | **(N=18)** | **(N=5)** | **(N=9)** | **(N=38)** |  |
| **Q1. I enjoy the work I do relating to MAID.** |  |  |  |  |  |  |
| 3 | 0 (0.00 %) | 0 (0.00 %) | 0 (0.00 %) | 1 (11.11 %) | 1 (2.63 %) | 0.22 |
| 4 | 2 (33.33 %) | 4 (22.22 %) | 1 (20.00 %) | 5 (55.56 %) | 12 (31.58 %) |  |
| 5 | 4 (66.67 %) | 14 (77.78 %) | 4 (80.00 %) | 3 (33.33 %) | 25 (65.79 %) |  |
| **Q2. My work with MAID is draining.** |  |  |  |  |  |  |
| 1 | 0 (0.00 %) | 3 (16.67 %) | 0 (0.00 %) | 0 (0.00 %) | 3 (7.89 %) | 0.42 |
| 2 | 3 (50.00 %) | 8 (44.44 %) | 1 (20.00 %) | 3 (33.33 %) | 15 (39.47 %) |  |
| 3 | 2 (33.33 %) | 2 (11.11 %) | 0 (0.00 %) | 3 (33.33 %) | 7 (18.42 %) |  |
| 4 | 1 (16.67 %) | 5 (27.78 %) | 4 (80.00 %) | 3 (33.33 %) | 13 (34.21 %) |  |
| **Q3. I often experience distress relating to my work with MAID.** |  |  |  |  |  |  |
| 1 | 0 (0.00 %) | 6 (33.33 %) | 0 (0.00 %) | 1 (11.11 %) | 7 (18.42 %) | 0.29 |
| 2 | 4 (66.67 %) | 8 (44.44 %) | 3 (60.00 %) | 7 (77.78 %) | 22 (57.89 %) |  |
| 3 | 1 (16.67 %) | 4 (22.22 %) | 1 (20.00 %) | 1 (11.11 %) | 7 (18.42 %) |  |
| 4 | 1 (16.67 %) | 0 (0.00 %) | 1 (20.00 %) | 0 (0.00 %) | 2 (5.26 %) |  |
| **Q4. My work with MAID has caused me to feel burnt out.** |  |  |  |  |  |  |
| 1 | 3 (50.00 %) | 11 (61.11 %) | 0 (0.00 %) | 0 (0.00 %) | 14 (36.84 %) | 0.006 |
| 2 | 3 (50.00 %) | 6 (33.33 %) | 5 (100.00 %) | 7 (77.78 %) | 21 (55.26 %) |  |
| 3 | 0 (0.00 %) | 1 (5.56 %) | 0 (0.00 %) | 1 (11.11 %) | 2 (5.26 %) |  |
| 4 | 0 (0.00 %) | 0 (0.00 %) | 0 (0.00 %) | 1 (11.11 %) | 1 (2.63 %) |  |
| **Q5. I believe my work with MAID has a negative impact on the way my colleagues view me.** |  |  |  |  |  |  |
| 1 | 1 (16.67 %) | 8 (44.44 %) | 2 (40.00 %) | 3 (33.33 %) | 14 (36.84 %) | 0.20 |
| 2 | 3 (50.00 %) | 7 (38.89 %) | 0 (0.00 %) | 2 (22.22 %) | 12 (31.58 %) |  |
| 3 | 1 (16.67 %) | 2 (11.11 %) | 3 (60.00 %) | 4 (44.44 %) | 10 (26.32 %) |  |
| 4 | 0 (0.00 %) | 1 (5.56 %) | 0 (0.00 %) | 0 (0.00 %) | 1 (2.63 %) |  |
| 5 | 1 (16.67 %) | 0 (0.00 %) | 0 (0.00 %) | 0 (0.00 %) | 1 (2.63 %) |  |
| **Q6. My experience providing MAID has had a negative impact on the way I view MAID** |  |  |  |  |  |  |
| 1 | 5 (83.33 %) | 16 (88.89 %) | 3 (60.00 %) | 5 (55.56 %) | 29 (76.32 %) | 0.26 |
| 2 | 1 (16.67 %) | 2 (11.11 %) | 2 (40.00 %) | 3 (33.33 %) | 8 (21.05 %) |  |
| 4 | 0 (0.00 %) | 0 (0.00 %) | 0 (0.00 %) | 1 (11.11 %) | 1 (2.63 %) |  |
| **Q7. My experience providing MAID has made me less comfortable with future plans to expand access.** |  |  |  |  |  |  |
| 1 | 4 (66.67 %) | 6 (33.33 %) | 2 (40.00 %) | 3 (33.33 %) | 15 (39.47 %) | 0.16 |
| 2 | 2 (33.33 %) | 7 (38.89 %) | 1 (20.00 %) | 0 (0.00 %) | 10 (26.32 %) |  |
| 3 | 0 (0.00 %) | 1 (5.56 %) | 0 (0.00 %) | 1 (11.11 %) | 2 (5.26 %) |  |
| 4 | 0 (0.00 %) | 2 (11.11 %) | 1 (20.00 %) | 5 (55.56 %) | 8 (21.05 %) |  |
| 5 | 0 (0.00 %) | 2 (11.11 %) | 1 (20.00 %) | 0 (0.00 %) | 3 (7.89 %) |  |
| **Q8. My experience providing MAID has made me more concerned about how MAiD is being provided elsewhere in the community.** |  |  |  |  |  |  |
| 1 | 3 (50.00 %) | 6 (33.33 %) | 2 (40.00 %) | 2 (22.22 %) | 13 (34.21 %) | 0.20 |
| 2 | 2 (33.33 %) | 7 (38.89 %) | 0 (0.00 %) | 1 (11.11 %) | 10 (26.32 %) |  |
| 3 | 1 (16.67 %) | 1 (5.56 %) | 0 (0.00 %) | 1 (11.11 %) | 3 (7.89 %) |  |
| 4 | 0 (0.00 %) | 4 (22.22 %) | 1 (20.00 %) | 5 (55.56 %) | 10 (26.32 %) |  |
| 5 | 0 (0.00 %) | 0 (0.00 %) | 1 (20.00 %) | 0 (0.00 %) | 1 (2.63 %) |  |
| Missing | 0 (0%) | 0 (0%) | 1 (20.0%) | 0 (0%) | 1 (2.6%) |  |
| **Q9. I am concerned about potential stigma or judgement from the broader community due to my involvement with MAID.** |  |  |  |  |  |  |
| 1 | 2 (33.33 %) | 6 (33.33 %) | 2 (40.00 %) | 3 (33.33 %) | 13 (34.21 %) | 0.26 |
| 2 | 0 (0.00 %) | 4 (22.22 %) | 0 (0.00 %) | 5 (55.56 %) | 9 (23.68 %) |  |
| 3 | 2 (33.33 %) | 5 (27.78 %) | 1 (20.00 %) | 1 (11.11 %) | 9 (23.68 %) |  |
| 4 | 1 (16.67 %) | 3 (16.67 %) | 2 (40.00 %) | 0 (0.00 %) | 6 (15.79 %) |  |
| 5 | 1 (16.67 %) | 0 (0.00 %) | 0 (0.00 %) | 0 (0.00 %) | 1 (2.63 %) |  |
| **Q10. I cope well with the stress I experience directly related to my work with MAID.** |  |  |  |  |  |  |
| 1 | 1 (16.67 %) | 0 (0.00 %) | 0 (0.00 %) | 0 (0.00 %) | 1 (2.63 %) | 0.47 |
| 3 | 0 (0.00 %) | 1 (5.56 %) | 1 (20.00 %) | 1 (11.11 %) | 3 (7.89 %) |  |
| 4 | 4 (66.67 %) | 10 (55.56 %) | 3 (60.00 %) | 7 (77.78 %) | 24 (63.16 %) |  |
| 5 | 1 (16.7%) | 7 (38.9%) | 1 (20.0%) | 1 (11.1%) | 10 (26.3%) |  |
| **Q11. I have seriously considered discontinuing my work with MAID.** |  |  |  |  |  |  |
| 1 | 4 (66.67 %) | 15 (83.33 %) | 3 (60.00 %) | 2 (22.22 %) | 24 (63.16 %) | <0.001 |
| 2 | 1 (16.67 %) | 2 (11.11 %) | 0 (0.00 %) | 7 (77.78 %) | 10 (26.32 %) |  |
| 3 | 0 (0.00 %) | 1 (5.56 %) | 2 (40.00 %) | 0 (0.00 %) | 3 (7.89 %) |  |
| 4 | 1 (16.67 %) | 0 (0.00 %) | 0 (0.00 %) | 0 (0.00 %) | 1 (2.63 %) |  |
| **Q12. I believe MAID practitioners should be limited to the number of MAID cases they can perform per year.** |  |  |  |  |  |  |
| 1 | 1 (16.67 %) | 0 (0.00 %) | 0 (0.00 %) | 0 (0.00 %) | 1 (2.63 %) | 0.47 |
| 3 | 0 (0.00 %) | 1 (5.56 %) | 1 (20.00 %) | 1 (11.11 %) | 3 (7.89 %) |  |
| 4 | 4 (66.67 %) | 10 (55.56 %) | 3 (60.00 %) | 7 (77.78 %) | 24 (63.16 %) |  |
| 5 | 1 (16.67 %) | 7 (38.89 %) | 1 (20.00 %) | 1 (11.11 %) | 10 (26.32 %) |  |
| **Q13. I have individuals I can go to who are clinically and ethically knowledgeable about MAID as a support or to discuss difficult cases.** |  |  |  |  |  |  |
| 1 | 1 (16.67 %) | 0 (0.00 %) | 0 (0.00 %) | 0 (0.00 %) | 1 (2.63 %) | 0.47 |
| 4 | 3 (50.00 %) | 7 (38.89 %) | 1 (20.00 %) | 4 (44.44 %) | 15 (39.47 %) |  |
| 5 | 2 (33.33 %) | 11 (61.11 %) | 4 (80.00 %) | 5 (55.56 %) | 22 (57.89 %) |  |
| **Q14. I feel well compensated for my work involving MAID.** |  |  |  |  |  |  |
| 1 | 2 (33.33 %) | 1 (5.56 %) | 4 (80.00 %) | 1 (11.11 %) | 8 (21.05 %) | 0.07 |
| 2 | 2 (33.33 %) | 6 (33.33 %) | 1 (20.00 %) | 1 (11.11 %) | 10 (26.32 %) |  |
| 3 | 0 (0.00 %) | 4 (22.22 %) | 0 (0.00 %) | 2 (22.22 %) | 6 (15.79 %) |  |
| 4 | 1 (16.67 %) | 5 (27.78 %) | 0 (0.00 %) | 5 (55.56 %) | 11 (28.95 %) |  |
| 5 | 1 (16.67 %) | 2 (11.11 %) | 0 (0.00 %) | 0 (0.00 %) | 3 (7.89 %) |  |
| **Q15. If I was better compensated for MAID work I would commit more time to it.** |  |  |  |  |  |  |
| 1 | 0 (0.00 %) | 6 (33.33 %) | 0 (0.00 %) | 0 (0.00 %) | 6 (15.79 %) | 0.22 |
| 2 | 4 (66.67 %) | 5 (27.78 %) | 1 (20.00 %) | 5 (55.56 %) | 15 (39.47 %) |  |
| 3 | 1 (16.67 %) | 2 (11.11 %) | 1 (20.00 %) | 1 (11.11 %) | 5 (13.16 %) |  |
| 4 | 1 (16.67 %) | 4 (22.22 %) | 1 (20.00 %) | 3 (33.33 %) | 9 (23.68 %) |  |
| 5 | 0 (0.00 %) | 1 (5.56 %) | 2 (40.00 %) | 0 (0.00 %) | 3 (7.89 %) |  |
| **Q16. I received a sufficient amount of training on the emotional / psychological impact of MAID.** |  |  |  |  |  |  |
| 1 | 0 (0.00 %) | 0 (0.00 %) | 0 (0.00 %) | 1 (11.11 %) | 1 (2.63 %) | 0.10 |
| 2 | 3 (50.00 %) | 1 (5.56 %) | 2 (40.00 %) | 3 (33.33 %) | 9 (23.68 %) |  |
| 3 | 1 (16.67 %) | 10 (55.56 %) | 0 (0.00 %) | 3 (33.33 %) | 14 (36.84 %) |  |
| 4 | 2 (33.33 %) | 5 (27.78 %) | 2 (40.00 %) | 1 (11.11 %) | 10 (26.32 %) |  |
| 5 | 0 (0.00 %) | 2 (11.11 %) | 1 (20.00 %) | 1 (11.11 %) | 4 (10.53 %) |  |
| **Q17. Additional training on the emotional / psychological aspects of MAID would be beneficial for me.** |  |  |  |  |  |  |
| 1 | 0 (0.00 %) | 2 (11.11 %) | 0 (0.00 %) | 1 (11.11 %) | 3 (7.89 %) | 0.59 |
| 2 | 1 (16.67 %) | 8 (44.44 %) | 1 (20.00 %) | 1 (11.11 %) | 11 (28.95 %) |  |
| 3 | 3 (50.00 %) | 3 (16.67 %) | 1 (20.00 %) | 4 (44.44 %) | 11 (28.95 %) |  |
| 4 | 2 (33.33 %) | 5 (27.78 %) | 3 (60.00 %) | 3 (33.33 %) | 13 (34.21 %) |  |
| **Q18. Ideally, I would complete fewer cases of MAID.** |  |  |  |  |  |  |
| 1 | 0 (0.00 %) | 9 (50.00 %) | 3 (60.00 %) | 1 (11.11 %) | 13 (34.21 %) | 0.08 |
| 2 | 4 (66.67 %) | 7 (38.89 %) | 1 (20.00 %) | 7 (77.78 %) | 19 (50.00 %) |  |
| 3 | 1 (16.67 %) | 1 (5.56 %) | 1 (20.00 %) | 1 (11.11 %) | 4 (10.53 %) |  |
| 4 | 1 (16.67 %) | 1 (5.56 %) | 0 (0.00 %) | 0 (0.00 %) | 2 (5.26 %) |  |
| **Q19. I feel my current workload relating to MAID is sustainable in terms of its impact on my wellbeing over time.** |  |  |  |  |  |  |
| 1 | 0 (0.00 %) | 1 (5.56 %) | 0 (0.00 %) | 0 (0.00 %) | 1 (2.63 %) | 0.003 |
| 2 | 1 (16.67 %) | 0 (0.00 %) | 3 (60.00 %) | 1 (11.11 %) | 5 (13.16 %) |  |
| 3 | 1 (16.67 %) | 2 (11.11 %) | 1 (20.00 %) | 0 (0.00 %) | 4 (10.53 %) |  |
| 4 | 4 (66.67 %) | 7 (38.89 %) | 1 (20.00 %) | 8 (88.89 %) | 20 (52.63 %) |  |
| 5 | 0 (0.00 %) | 8 (44.44 %) | 0 (0.00 %) | 0 (0.00 %) | 8 (21.05 %) |  |
| **Q20. I am aware of other healthcare workers who have removed themselves from their work with MAID due to burnout (or other reasons relating to emotional / psychological distress).** |  |  |  |  |  |  |
| 1 | 0 (0.00 %) | 5 (27.78 %) | 0 (0.00 %) | 0 (0.00 %) | 5 (13.16 %) | 0.27 |
| 2 | 1 (16.67 %) | 6 (33.33 %) | 2 (40.00 %) | 2 (22.22 %) | 11 (28.95 %) |  |
| 3 | 1 (16.67 %) | 3 (16.67 %) | 2 (40.00 %) | 3 (33.33 %) | 9 (23.68 %) |  |
| 4 | 2 (33.33 %) | 4 (22.22 %) | 1 (20.00 %) | 4 (44.44 %) | 11 (28.95 %) |  |
| 5 | 2 (33.33 %) | 0 (0.00 %) | 0 (0.00 %) | 0 (0.00 %) | 2 (5.26 %) |  |

**Table 2. MAID questionnaire responses analyzed by specialty using Fisher´s exact test. 1 = strongly disagree, 2 = disagree, 3 = neutral, 4 = agree, 5 = strongly agree.**

|  | **Track 1 Only** | **Track 2** | **Total** | **P-value** |
| --- | --- | --- | --- | --- |
|  | **(N=14)** | **(N=24)** | **(N=38)** |  |
| **Q1. I enjoy the work I do relating to MAID.** |  |  |  |  |
| 3 | 1 (7.14 %) | 0 (0.00 %) | 1 (2.63 %) | 0.005 |
| 4 | 8 (57.14 %) | 4 (16.67 %) | 12 (31.58 %) |  |
| 5 | 5 (35.71 %) | 20 (83.33 %) | 25 (65.79 %) |  |
| **Q2. My work with MAID is draining.** |  |  |  |  |
| 1 | 2 (14.29 %) | 1 (4.17 %) | 3 (7.89 %) | 0.34 |
| 2 | 4 (28.57 %) | 11 (45.83 %) | 15 (39.47 %) |  |
| 3 | 4 (28.57 %) | 3 (12.50 %) | 7 (18.42 %) |  |
| 4 | 4 (28.57 %) | 9 (37.50 %) | 13 (34.21 %) |  |
| **Q3. I often experience distress relating to my work with MAID.** |  |  |  |  |
| 1 | 2 (14.29 %) | 5 (20.83 %) | 7 (18.42 %) | 0.90 |
| 2 | 9 (64.29 %) | 13 (54.17 %) | 22 (57.89 %) |  |
| 3 | 2 (14.29 %) | 5 (20.83 %) | 7 (18.42 %) |  |
| 4 | 1 (7.14 %) | 1 (4.17 %) | 2 (5.26 %) |  |
| **Q4. My work with MAID has caused me to feel burnt out.** |  |  |  |  |
| 1 | 4 (28.57 %) | 10 (41.67 %) | 14 (36.84 %) | 0.24 |
| 2 | 8 (57.14 %) | 13 (54.17 %) | 21 (55.26 %) |  |
| 3 | 2 (14.29 %) | 0 (0.00 %) | 2 (5.26 %) |  |
| 4 | 0 (0.00 %) | 1 (4.17 %) | 1 (2.63 %) |  |
| **Q5. I believe my work with MAID has a negative impact on the way my colleagues view me.** |  |  |  |  |
| 1 | 2 (14.29 %) | 12 (50.00 %) | 14 (36.84 %) | 0.06 |
| 2 | 5 (35.71 %) | 7 (29.17 %) | 12 (31.58 %) |  |
| 3 | 6 (42.86 %) | 4 (16.67 %) | 10 (26.32 %) |  |
| 4 | 1 (7.14 %) | 0 (0.00 %) | 1 (2.63 %) |  |
| 5 | 0 (0.00 %) | 1 (4.17 %) | 1 (2.63 %) |  |
| **Q6. My experience providing MAID has had a negative impact on the way I view MAID** |  |  |  |  |
| 1 | 8 (57.14 %) | 21 (87.50 %) | 29 (76.32 %) | 0.06 |
| 2 | 5 (35.71 %) | 3 (12.50 %) | 8 (21.05 %) |  |
| 4 | 1 (7.14 %) | 0 (0.00 %) | 1 (2.63 %) |  |
| **Q7. My experience providing MAID has made me less comfortable with future plans to expand access.** |  |  |  |  |
| 1 | 2 (14.29 %) | 13 (54.17 %) | 15 (39.47 %) | 0.02 |
| 2 | 3 (21.43 %) | 7 (29.17 %) | 10 (26.32 %) |  |
| 3 | 2 (14.29 %) | 0 (0.00 %) | 2 (5.26 %) |  |
| 4 | 5 (35.71 %) | 3 (12.50 %) | 8 (21.05 %) |  |
| 5 | 2 (14.29 %) | 1 (4.17 %) | 3 (7.89 %) |  |
| **Q8. My experience providing MAID has made me more concerned about how MAiD is being provided elsewhere in the community.** |  |  |  |  |
| 1 | 1 (7.14 %) | 12 (50.00 %) | 13 (34.21 %) | 0.03 |
| 2 | 5 (35.71 %) | 5 (20.83 %) | 10 (26.32 %) |  |
| 3 | 2 (14.29 %) | 1 (4.17 %) | 3 (7.89 %) |  |
| 4 | 5 (35.71 %) | 5 (20.83 %) | 10 (26.32 %) |  |
| 5 | 1 (7.14 %) | 0 (0.00 %) | 1 (2.63 %) |  |
| Missing | 0 (0%) | 1 (4.2%) | 1 (2.6%) |  |
| **Q9. I am concerned about potential stigma or judgement from the broader community due to my involvement with MAID.** |  |  |  |  |
| 1 | 3 (21.43 %) | 10 (41.67 %) | 13 (34.21 %) | 0.54 |
| 2 | 5 (35.71 %) | 4 (16.67 %) | 9 (23.68 %) |  |
| 3 | 4 (28.57 %) | 5 (20.83 %) | 9 (23.68 %) |  |
| 4 | 2 (14.29 %) | 4 (16.67 %) | 6 (15.79 %) |  |
| 5 | 0 (0.00 %) | 1 (4.17 %) | 1 (2.63 %) |  |
| **Q10. I cope well with the stress I experience directly related to my work with MAID.** |  |  |  |  |
| 1 | 0 (0.00 %) | 1 (4.17 %) | 1 (2.63 %) | 0.08 |
| 3 | 3 (21.43 %) | 0 (0.00 %) | 3 (7.89 %) |  |
| 4 | 7 (50.00 %) | 17 (70.83 %) | 24 (63.16 %) |  |
| 5 | 4 (28.6%) | 6 (25.0%) | 10 (26.3%) |  |
| **Q11. I have seriously considered discontinuing my work with MAID.** |  |  |  |  |
| 1 | 5 (35.71 %) | 19 (79.17 %) | 24 (63.16 %) | 0.01 |
| 2 | 7 (50.00 %) | 3 (12.50 %) | 10 (26.32 %) |  |
| 3 | 2 (14.29 %) | 1 (4.17 %) | 3 (7.89 %) |  |
| 4 | 0 (0.00 %) | 1 (4.17 %) | 1 (2.63 %) |  |
| **Q12. I believe MAID practitioners should be limited to the number of MAID cases they can perform per year.** |  |  |  |  |
| 1 | 0 (0.00 %) | 1 (4.17 %) | 1 (2.63 %) | 0.08 |
| 3 | 3 (21.43 %) | 0 (0.00 %) | 3 (7.89 %) |  |
| 4 | 7 (50.00 %) | 17 (70.83 %) | 24 (63.16 %) |  |
| 5 | 4 (28.57 %) | 6 (25.00 %) | 10 (26.32 %) |  |
| **Q13. I have individuals I can go to who are clinically and ethically knowledgeable about MAID as a support or to discuss difficult cases.** |  |  |  |  |
| 1 | 0 (0.00 %) | 1 (4.17 %) | 1 (2.63 %) | 0.68 |
| 4 | 7 (50.00 %) | 8 (33.33 %) | 15 (39.47 %) |  |
| 5 | 7 (50.00 %) | 15 (62.50 %) | 22 (57.89 %) |  |
| **Q14. I feel well compensated for my work involving MAID.** |  |  |  |  |
| 1 | 1 (7.14 %) | 7 (29.17 %) | 8 (21.05 %) | 0.25 |
| 2 | 4 (28.57 %) | 6 (25.00 %) | 10 (26.32 %) |  |
| 3 | 3 (21.43 %) | 3 (12.50 %) | 6 (15.79 %) |  |
| 4 | 6 (42.86 %) | 5 (20.83 %) | 11 (28.95 %) |  |
| 5 | 0 (0.00 %) | 3 (12.50 %) | 3 (7.89 %) |  |
| **Q15. If I was better compensated for MAID work I would commit more time to it.** |  |  |  |  |
| 1 | 1 (7.14 %) | 5 (20.83 %) | 6 (15.79 %) | 0.14 |
| 2 | 8 (57.14 %) | 7 (29.17 %) | 15 (39.47 %) |  |
| 3 | 0 (0.00 %) | 5 (20.83 %) | 5 (13.16 %) |  |
| 4 | 3 (21.43 %) | 6 (25.00 %) | 9 (23.68 %) |  |
| 5 | 2 (14.29 %) | 1 (4.17 %) | 3 (7.89 %) |  |
| **Q16. I received a sufficient amount of training on the emotional / psychological impact of MAID.** |  |  |  |  |
| 1 | 1 (7.14 %) | 0 (0.00 %) | 1 (2.63 %) | 0.29 |
| 2 | 3 (21.43 %) | 6 (25.00 %) | 9 (23.68 %) |  |
| 3 | 7 (50.00 %) | 7 (29.17 %) | 14 (36.84 %) |  |
| 4 | 3 (21.43 %) | 7 (29.17 %) | 10 (26.32 %) |  |
| 5 | 0 (0.00 %) | 4 (16.67 %) | 4 (10.53 %) |  |
| **Q17. Additional training on the emotional / psychological aspects of MAID would be beneficial for me.** |  |  |  |  |
| 1 | 2 (14.29 %) | 1 (4.17 %) | 3 (7.89 %) | 0.32 |
| 2 | 2 (14.29 %) | 9 (37.50 %) | 11 (28.95 %) |  |
| 3 | 4 (28.57 %) | 7 (29.17 %) | 11 (28.95 %) |  |
| 4 | 6 (42.86 %) | 7 (29.17 %) | 13 (34.21 %) |  |
| **Q18. Ideally, I would complete fewer cases of MAID.** |  |  |  |  |
| 1 | 3 (21.43 %) | 10 (41.67 %) | 13 (34.21 %) | 0.42 |
| 2 | 9 (64.29 %) | 10 (41.67 %) | 19 (50.00 %) |  |
| 3 | 2 (14.29 %) | 2 (8.33 %) | 4 (10.53 %) |  |
| 4 | 0 (0.00 %) | 2 (8.33 %) | 2 (5.26 %) |  |
| **Q19. I feel my current workload relating to MAID is sustainable in terms of its impact on my wellbeing over time.** |  |  |  |  |
| 1 | 1 (7.14 %) | 0 (0.00 %) | 1 (2.63 %) | 0.13 |
| 2 | 0 (0.00 %) | 5 (20.83 %) | 5 (13.16 %) |  |
| 3 | 1 (7.14 %) | 3 (12.50 %) | 4 (10.53 %) |  |
| 4 | 10 (71.43 %) | 10 (41.67 %) | 20 (52.63 %) |  |
| 5 | 2 (14.29 %) | 6 (25.00 %) | 8 (21.05 %) |  |
| **Q20. I am aware of other healthcare workers who have removed themselves from their work with MAID due to burnout (or other reasons relating to emotional / psychological distress).** |  |  |  |  |
| 1 | 2 (14.29 %) | 3 (12.50 %) | 5 (13.16 %) | 0.82 |
| 2 | 5 (35.71 %) | 6 (25.00 %) | 11 (28.95 %) |  |
| 3 | 4 (28.57 %) | 5 (20.83 %) | 9 (23.68 %) |  |
| 4 | 3 (21.43 %) | 8 (33.33 %) | 11 (28.95 %) |  |
| 5 | 0 (0.00 %) | 2 (8.33 %) | 2 (5.26 %) |  |

**Table 3. MAID questionnaire responses analyzed using Fisher´s exact test comparing those who complete solely Track 1 cases vs those who complete Track 2 cases. 1 = strongly disagree, 2 = disagree, 3 = neutral, 4 = agree, 5 = strongly agree.**

|  | **3** | **4** | **5** | **Total** | **P-value** |
| --- | --- | --- | --- | --- | --- |
|  | **(N=1)** | **(N=12)** | **(N=25)** | **(N=38)** |  |
| **Years as healthcare provider** |  |  |  |  |  |
| Median (IQR) | 25 (± 0) | 19 (± 7.3) | 27 (± 19) | 22 (± 16) | 0.58 |
| **Years as MAiD provider** |  |  |  |  |  |
| Median (IQR) | 8.0 (± 0) | 7.5 (± 3.0) | 6.0 (± 3.0) | 7.0 (± 3.0) | 0.21 |
| **Number of MAiD provisions in last 12 months** |  |  |  |  |  |
| Median (IQR) | 12 (± 0) | 9.0 (± 7.3) | 15 (± 20) | 12 (± 22) | 0.13 |
| **Number of hours spent on MAiD work per month** |  |  |  |  |  |
| Median (IQR) | 4.0 (± 0) | 7.0 (± 5.5) | 20 (± 40) | 15 (± 27) | 0.02 |

**Table 4. Provider experience characteristics according to questionnaire response to “I enjoy the work I do relating to MAID”. Analysis completed using Kruskal-Wallis test.**

|  | **1** | **2** | **3** | **4** | **Total** | **P-value** |
| --- | --- | --- | --- | --- | --- | --- |
|  | **(N=3)** | **(N=15)** | **(N=7)** | **(N=13)** | **(N=38)** |  |
| **Years as healthcare provider** |  |  |  |  |  |  |
| Median (IQR) | 31 (± 18) | 27 (± 16) | 16 (± 12) | 21 (± 11) | 22 (± 16) | 0.62 |
| **Years as MAiD provider** |  |  |  |  |  |  |
| Median (IQR) | 3.0 (± 1.5) | 7.0 (± 4.0) | 7.0 (± 2.5) | 7.0 (± 3.0) | 7.0 (± 3.0) | 0.23 |
| **Number of MAiD provisions in last 12 months** |  |  |  |  |  |  |
| Median (IQR) | 5.0 (± 2.5) | 15 (± 9.0) | 6.0 (± 14) | 25 (± 20) | 12 (± 22) | 0.18 |
| **Number of hours spent on MAiD work per month** |  |  |  |  |  |  |
| Median (IQR) | 4.0 (± 13) | 20 (± 36) | 5.0 (± 5.8) | 15 (± 32) | 15 (± 27) | 0.07 |

**Table 5. Provider experience characteristics according to questionnaire response to “My work with MAID is draining”. Analysis completed using Kruskal-Wallis test.**

|  | **1** | **2** | **3** | **4** | **Total** | **P-value** |
| --- | --- | --- | --- | --- | --- | --- |
|  | **(N=7)** | **(N=22)** | **(N=7)** | **(N=2)** | **(N=38)** |  |
| **Years as healthcare provider** |  |  |  |  |  |  |
| Median (IQR) | 28 (± 24) | 24 (± 16) | 19 (± 4.5) | 15 (± 2.5) | 22 (± 16) | 0.47 |
| **Years as MAiD provider** |  |  |  |  |  |  |
| Median (IQR) | 7.0 (± 5.0) | 7.0 (± 3.0) | 6.0 (± 3.5) | 5.5 (± 0.50) | 7.0 (± 3.0) | 0.80 |
| **Number of MAiD provisions in last 12 months** |  |  |  |  |  |  |
| Median (IQR) | 15 (± 20) | 14 (± 22) | 10 (± 4.0) | 16 (± 14) | 12 (± 22) | 0.70 |
| **Number of hours spent on MAiD work per month** |  |  |  |  |  |  |
| Median (IQR) | 28 (± 23) | 17 (± 37) | 8.0 (± 7.5) | 8.5 (± 6.5) | 15 (± 27) | 0.19 |

**Table 6. Provider experience characteristics according to questionnaire response to “I often experience distress relating to my work with MAID”. Analysis completed using Kruskal-Wallis test.**

|  | **1** | **2** | **3** | **4** | **Total** | **P-value** |
| --- | --- | --- | --- | --- | --- | --- |
|  | **(N=14)** | **(N=21)** | **(N=2)** | **(N=1)** | **(N=38)** |  |
| **Years as healthcare provider** |  |  |  |  |  |  |
| Median (IQR) | 29 (± 14) | 22 (± 14) | 11 (± 8.5) | 19 (± 0) | 22 (± 16) | 0.32 |
| **Years as MAiD provider** |  |  |  |  |  |  |
| Median (IQR) | 7.0 (± 3.5) | 7.0 (± 3.0) | 3.5 (± 1.5) | 5.0 (± 0) | 7.0 (± 3.0) | 0.29 |
| **Number of MAiD provisions in last 12 months** |  |  |  |  |  |  |
| Median (IQR) | 11 (± 12) | 12 (± 24) | 19 (± 6.5) | 30 (± 0) | 12 (± 22) | 0.70 |
| **Number of hours spent on MAiD work per month** |  |  |  |  |  |  |
| Median (IQR) | 17 (± 22) | 15 (± 35) | 10 (± 0) | 40 (± 0) | 15 (± 27) | 0.74 |

**Table 7. Provider experience characteristics according to questionnaire response to “My work with MAID has caused me to feel burnt out”. Analysis completed using Kruskal-Wallis test.**

|  | **1** | **2** | **3** | **4** | **5** | **Total** | **P-value** |
| --- | --- | --- | --- | --- | --- | --- | --- |
|  | **(N=14)** | **(N=12)** | **(N=10)** | **(N=1)** | **(N=1)** | **(N=38)** |  |
| **Years as healthcare provider** |  |  |  |  |  |  |  |
| Median (IQR) | 29 (± 16) | 23 (± 18) | 19 (± 6.8) | 4.0 (± 0) | 28 (± 0) | 22 (± 16) | 0.23 |
| **Years as MAiD provider** |  |  |  |  |  |  |  |
| Median (IQR) | 6.5 (± 1.8) | 7.0 (± 3.3) | 6.0 (± 3.0) | 2.0 (± 0) | 3.0 (± 0) | 7.0 (± 3.0) | 0.37 |
| **Number of MAiD provisions in last 12 months** |  |  |  |  |  |  |  |
| Median (IQR) | 18 (± 20) | 9.0 (± 14) | 14 (± 12) | 5.0 (± 0) | 20 (± 0) | 12 (± 22) | 0.39 |
| **Number of hours spent on MAiD work per month** |  |  |  |  |  |  |  |
| Median (IQR) | 34 (± 42) | 12 (± 20) | 10 (± 7.0) | 4.0 (± 0) | 20 (± 0) | 15 (± 27) | 0.37 |

**Table 8. Provider experience characteristics according to questionnaire response to “I believe my work with MAID has a negative impact on the way my colleagues view me”. Analysis completed using Kruskal-Wallis test.**

|  | **1** | **2** | **4** | **Total** | **P-value** |
| --- | --- | --- | --- | --- | --- |
|  | **(N=29)** | **(N=8)** | **(N=1)** | **(N=38)** |  |
| **Years as healthcare provider** |  |  |  |  |  |
| Median (IQR) | 27 (± 17) | 19 (± 5.5) | 25 (± 0) | 22 (± 16) | 0.33 |
| **Years as MAiD provider** |  |  |  |  |  |
| Median (IQR) | 7.0 (± 3.0) | 5.5 (± 3.0) | 8.0 (± 0) | 7.0 (± 3.0) | 0.45 |
| **Number of MAiD provisions in last 12 months** |  |  |  |  |  |
| Median (IQR) | 12 (± 24) | 11 (± 17) | 12 (± 0) | 12 (± 22) | 0.99 |
| **Number of hours spent on MAiD work per month** |  |  |  |  |  |
| Median (IQR) | 20 (± 37) | 10 (± 8.0) | 4.0 (± 0) | 15 (± 27) | 0.14 |

**Table 9. Provider experience characteristics according to questionnaire response to “My experience providing MAID has had a negative impact on the way I view MAID”. Analysis completed using Kruskal-Wallis test.**

|  | **1** | **2** | **3** | **4** | **5** | **Total** | **P-value** |
| --- | --- | --- | --- | --- | --- | --- | --- |
|  | **(N=15)** | **(N=10)** | **(N=2)** | **(N=8)** | **(N=3)** | **(N=38)** |  |
| **Years as healthcare provider** |  |  |  |  |  |  |  |
| Median (IQR) | 19 (± 24) | 29 (± 9.8) | 19 (± 6.5) | 21 (± 12) | 21 (± 6.5) | 22 (± 16) | 0.46 |
| **Years as MAiD provider** |  |  |  |  |  |  |  |
| Median (IQR) | 6.0 (± 2.5) | 7.0 (± 2.3) | 3.0 (± 2.0) | 7.5 (± 5.3) | 7.0 (± 1.5) | 7.0 (± 3.0) | 0.38 |
| **Number of MAiD provisions in last 12 months** |  |  |  |  |  |  |  |
| Median (IQR) | 20 (± 22) | 12 (± 7.5) | 7.0 (± 3.0) | 14 (± 19) | 10 (± 11) | 12 (± 22) | 0.63 |
| **Number of hours spent on MAiD work per month** |  |  |  |  |  |  |  |
| Median (IQR) | 20 (± 48) | 20 (± 30) | 3.0 (± 0) | 9.0 (± 8.8) | 15 (± 8.5) | 15 (± 27) | 0.34 |

**Table 10. Provider experience characteristics according to questionnaire response to “My experience providing MAID has made me less comfortable with future plans to expand access”. Analysis completed using Kruskal-Wallis test.**

|  | **1** | **2** | **3** | **4** | **5** | **Total** | **P-value** |
| --- | --- | --- | --- | --- | --- | --- | --- |
|  | **(N=13)** | **(N=10)** | **(N=3)** | **(N=10)** | **(N=1)** | **(N=37)** |  |
| **Years as healthcare provider** |  |  |  |  |  |  |  |
| Median (IQR) | 22 (± 18) | 19 (± 12) | 40 (± 15) | 25 (± 6.0) | 17 (± 0) | 22 (± 16) | 0.40 |
| **Years as MAiD provider** |  |  |  |  |  |  |  |
| Median (IQR) | 7.0 (± 1.0) | 5.0 (± 3.5) | 8.0 (± 2.5) | 7.5 (± 2.5) | 5.0 (± 0) | 7.0 (± 3.0) | 0.35 |
| **Number of MAiD provisions in last 12 months** |  |  |  |  |  |  |  |
| Median (IQR) | 12 (± 24) | 12 (± 14) | 4.0 (± 130) | 14 (± 13) | 30 (± 0) | 12 (± 19) | 0.69 |
| **Number of hours spent on MAiD work per month** |  |  |  |  |  |  |  |
| Median (IQR) | 20 (± 36) | 9.0 (± 7.8) | 77 (± 73) | 12 (± 20) | 15 (± 0) | 15 (± 24) | 0.65 |

**Table 11. Provider experience characteristics according to questionnaire response to “My experience providing MAID has made me more concerned about how MAiD is being provided elsewhere in the community”. Analysis completed using Kruskal-Wallis test.**

|  | **1** | **2** | **3** | **4** | **5** | **Total** | **P-value** |
| --- | --- | --- | --- | --- | --- | --- | --- |
|  | **(N=13)** | **(N=9)** | **(N=9)** | **(N=6)** | **(N=1)** | **(N=38)** |  |
| **Years as healthcare provider** |  |  |  |  |  |  |  |
| Median (IQR) | 19 (± 23) | 25 (± 13) | 21 (± 11) | 21 (± 9.0) | 28 (± 0) | 22 (± 16) | 0.96 |
| **Years as MAiD provider** |  |  |  |  |  |  |  |
| Median (IQR) | 7.0 (± 2.0) | 8.0 (± 1.0) | 7.0 (± 3.0) | 5.0 (± 1.5) | 3.0 (± 0) | 7.0 (± 3.0) | 0.34 |
| **Number of MAiD provisions in last 12 months** |  |  |  |  |  |  |  |
| Median (IQR) | 25 (± 34) | 12 (± 13) | 10 (± 4.0) | 10 (± 4.5) | 20 (± 0) | 12 (± 22) | 0.42 |
| **Number of hours spent on MAiD work per month** |  |  |  |  |  |  |  |
| Median (IQR) | 40 (± 54) | 10 (± 11) | 15 (± 16) | 12 (± 15) | 20 (± 0) | 15 (± 27) | 0.34 |

**Table 12. Provider experience characteristics according to questionnaire response to “I am concerned about potential stigma or judgement from the broader community due to my involvement with MAID”. Analysis completed using Kruskal-Wallis test.**

|  | **1** | **3** | **4** | **5** | **Total** | **P-value** |
| --- | --- | --- | --- | --- | --- | --- |
|  | **(N=1)** | **(N=3)** | **(N=24)** | **(N=10)** | **(N=38)** |  |
| **Years as healthcare provider** |  |  |  |  |  |  |
| Median (IQR) | 13 (± 0) | 17 (± 2.5) | 24 (± 11) | 27 (± 22) | 22 (± 16) | 0.49 |
| **Years as MAiD provider** |  |  |  |  |  |  |
| Median (IQR) | 7.0 (± 0) | 5.0 (± 1.0) | 7.0 (± 3.3) | 6.5 (± 2.0) | 7.0 (± 3.0) | 0.46 |
| **Number of MAiD provisions in last 12 months** |  |  |  |  |  |  |
| Median (IQR) | 4.0 (± 0) | 12 (± 15) | 12 (± 20) | 13 (± 9.8) | 12 (± 22) | 0.40 |
| **Number of hours spent on MAiD work per month** |  |  |  |  |  |  |
| Median (IQR) | 3.0 (± 0) | 10 (± 5.5) | 17 (± 28) | 19 (± 41) | 15 (± 27) | 0.46 |

**Table 13. Provider experience characteristics according to questionnaire response to “I cope well with the stress I experience directly related to my work with MAID”. Analysis completed using Kruskal-Wallis test.**

|  | **1** | **2** | **3** | **4** | **Total** | **P-value** |
| --- | --- | --- | --- | --- | --- | --- |
|  | **(N=24)** | **(N=10)** | **(N=3)** | **(N=1)** | **(N=38)** |  |
| **Years as healthcare provider** |  |  |  |  |  |  |
| Median (IQR) | 28 (± 16) | 20 (± 12) | 19 (± 1.0) | 9.0 (± 0) | 22 (± 16) | 0.08 |
| **Years as MAiD provider** |  |  |  |  |  |  |
| Median (IQR) | 7.0 (± 2.0) | 8.0 (± 2.8) | 5.0 (± 1.5) | 4.0 (± 0) | 7.0 (± 3.0) | 0.18 |
| **Number of MAiD provisions in last 12 months** |  |  |  |  |  |  |
| Median (IQR) | 14 (± 24) | 11 (± 16) | 12 (± 10) | 10 (± 0) | 12 (± 22) | 0.78 |
| **Number of hours spent on MAiD work per month** |  |  |  |  |  |  |
| Median (IQR) | 25 (± 43) | 6.0 (± 6.0) | 10 (± 3.5) | 20 (± 0) | 15 (± 27) | 0.07 |

**Table 14. Provider experience characteristics according to questionnaire response to “I have seriously considered discontinuing my work with MAID”. Analysis completed using Kruskal-Wallis test.**

|  | **1** | **3** | **4** | **5** | **Total** | **P-value** |
| --- | --- | --- | --- | --- | --- | --- |
|  | **(N=1)** | **(N=3)** | **(N=24)** | **(N=10)** | **(N=38)** |  |
| **Years as healthcare provider** |  |  |  |  |  |  |
| Median (IQR) | 13 (± 0) | 17 (± 2.5) | 24 (± 11) | 27 (± 22) | 22 (± 16) | 0.49 |
| **Years as MAiD provider** |  |  |  |  |  |  |
| Median (IQR) | 7.0 (± 0) | 5.0 (± 1.0) | 7.0 (± 3.3) | 6.5 (± 2.0) | 7.0 (± 3.0) | 0.46 |
| **Number of MAiD provisions in last 12 months** |  |  |  |  |  |  |
| Median (IQR) | 4.0 (± 0) | 12 (± 15) | 12 (± 20) | 13 (± 9.8) | 12 (± 22) | 0.40 |
| **Number of hours spent on MAiD work per month** |  |  |  |  |  |  |
| Median (IQR) | 3.0 (± 0) | 10 (± 5.5) | 17 (± 28) | 19 (± 41) | 15 (± 27) | 0.46 |

**Table 15. Provider experience characteristics according to questionnaire response to “I believe MAID practitioners should be limited to the number of MAID cases they can perform per year”. Analysis completed using Kruskal-Wallis test.**

|  | **1** | **4** | **5** | **Total** | **P-value** |
| --- | --- | --- | --- | --- | --- |
|  | **(N=1)** | **(N=15)** | **(N=22)** | **(N=38)** |  |
| **Years as healthcare provider** |  |  |  |  |  |
| Median (IQR) | 12 (± 0) | 22 (± 13) | 25 (± 13) | 22 (± 16) | 0.38 |
| **Years as MAiD provider** |  |  |  |  |  |
| Median (IQR) | 6.0 (± 0) | 7.0 (± 3.5) | 6.5 (± 3.0) | 7.0 (± 3.0) | 0.96 |
| **Number of MAiD provisions in last 12 months** |  |  |  |  |  |
| Median (IQR) | 2.0 (± 0) | 12 (± 19) | 14 (± 20) | 12 (± 22) | 0.21 |
| **Number of hours spent on MAiD work per month** |  |  |  |  |  |
| Median (IQR) | 2.0 (± 0) | 10 (± 12) | 20 (± 39) | 15 (± 27) | 0.16 |

**Table 16. Provider experience characteristics according to questionnaire response to “I have individuals I can go to who are clinically and ethically knowledgeable about MAID as a support or to discuss difficult cases”. Analysis completed using Kruskal-Wallis test.**

|  | **1** | **2** | **3** | **4** | **5** | **Total** | **P-value** |
| --- | --- | --- | --- | --- | --- | --- | --- |
|  | **(N=8)** | **(N=10)** | **(N=6)** | **(N=11)** | **(N=3)** | **(N=38)** |  |
| **Years as healthcare provider** |  |  |  |  |  |  |  |
| Median (IQR) | 18 (± 6.3) | 26 (± 13) | 28 (± 5.0) | 21 (± 17) | 40 (± 17) | 22 (± 16) | 0.27 |
| **Years as MAiD provider** |  |  |  |  |  |  |  |
| Median (IQR) | 6.5 (± 2.0) | 7.0 (± 2.8) | 6.5 (± 1.8) | 7.0 (± 5.5) | 4.0 (± 2.5) | 7.0 (± 3.0) | 0.82 |
| **Number of MAiD provisions in last 12 months** |  |  |  |  |  |  |  |
| Median (IQR) | 18 (± 22) | 14 (± 19) | 10 (± 4.5) | 12 (± 19) | 10 (± 120) | 12 (± 22) | 0.93 |
| **Number of hours spent on MAiD work per month** |  |  |  |  |  |  |  |
| Median (IQR) | 28 (± 46) | 10 (± 22) | 4.0 (± 17) | 12 (± 15) | 28 (± 65) | 15 (± 27) | 0.32 |

**Table 17. Provider experience characteristics according to questionnaire response to “I feel well compensated for my work involving MAID”. Analysis completed using Kruskal-Wallis test.**

|  | **1** | **2** | **3** | **4** | **5** | **Total** | **P-value** |
| --- | --- | --- | --- | --- | --- | --- | --- |
|  | **(N=6)** | **(N=15)** | **(N=5)** | **(N=9)** | **(N=3)** | **(N=38)** |  |
| **Years as healthcare provider** |  |  |  |  |  |  |  |
| Median (IQR) | 31 (± 6.3) | 25 (± 17) | 28 (± 13) | 19 (± 6.0) | 10 (± 6.5) | 22 (± 16) | 0.006 |
| **Years as MAiD provider** |  |  |  |  |  |  |  |
| Median (IQR) | 7.5 (± 1.0) | 6.0 (± 4.0) | 7.0 (± 1.0) | 5.0 (± 2.0) | 5.0 (± 2.5) | 7.0 (± 3.0) | 0.17 |
| **Number of MAiD provisions in last 12 months** |  |  |  |  |  |  |  |
| Median (IQR) | 14 (± 22) | 10 (± 16) | 20 (± 110) | 10 (± 19) | 15 (± 13) | 12 (± 22) | 0.16 |
| **Number of hours spent on MAiD work per month** |  |  |  |  |  |  |  |
| Median (IQR) | 33 (± 28) | 10 (± 17) | 50 (± 60) | 10 (± 6.0) | 15 (± 28) | 15 (± 27) | 0.10 |

**Table 18. Provider experience characteristics according to questionnaire response to “If I was better compensated for MAID work I would commit more time to it”. Analysis completed using Kruskal-Wallis test.**

|  | **1** | **2** | **3** | **4** | **5** | **Total** | **P-value** |
| --- | --- | --- | --- | --- | --- | --- | --- |
|  | **(N=1)** | **(N=9)** | **(N=14)** | **(N=10)** | **(N=4)** | **(N=38)** |  |
| **Years as healthcare provider** |  |  |  |  |  |  |  |
| Median (IQR) | 14 (± 0) | 19 (± 9.0) | 23 (± 11) | 28 (± 16) | 42 (± 8.0) | 22 (± 16) | 0.14 |
| **Years as MAiD provider** |  |  |  |  |  |  |  |
| Median (IQR) | 3.0 (± 0) | 7.0 (± 3.0) | 6.0 (± 3.5) | 6.5 (± 3.5) | 7.5 (± 1.3) | 7.0 (± 3.0) | 0.32 |
| **Number of MAiD provisions in last 12 months** |  |  |  |  |  |  |  |
| Median (IQR) | 0 (± 0) | 25 (± 24) | 12 (± 8.8) | 11 (± 8.8) | 180 (± 140) | 12 (± 22) | 0.13 |
| **Number of hours spent on MAiD work per month** |  |  |  |  |  |  |  |
| Median (IQR) | 4.0 (± 0) | 15 (± 15) | 15 (± 22) | 9.0 (± 15) | 120 (± 97) | 15 (± 27) | 0.34 |

**Table 19. Provider experience characteristics according to questionnaire response to “I received a sufficient amount of training on the emotional / psychological impact of MAID”. Analysis completed using Kruskal-Wallis test.**

|  | **1** | **2** | **3** | **4** | **Total** | **P-value** |
| --- | --- | --- | --- | --- | --- | --- |
|  | **(N=3)** | **(N=11)** | **(N=11)** | **(N=13)** | **(N=38)** |  |
| **Years as healthcare provider** |  |  |  |  |  |  |
| Median (IQR) | 22 (± 12) | 25 (± 9.5) | 28 (± 22) | 19 (± 10) | 22 (± 16) | 0.22 |
| **Years as MAiD provider** |  |  |  |  |  |  |
| Median (IQR) | 6.0 (± 3.0) | 7.0 (± 1.0) | 7.0 (± 2.5) | 5.0 (± 3.0) | 7.0 (± 3.0) | 0.05 |
| **Number of MAiD provisions in last 12 months** |  |  |  |  |  |  |
| Median (IQR) | 6.0 (± 5.0) | 12 (± 6.5) | 15 (± 38) | 10 (± 24) | 12 (± 22) | 0.71 |
| **Number of hours spent on MAiD work per month** |  |  |  |  |  |  |
| Median (IQR) | 4.0 (± 3.5) | 25 (± 34) | 20 (± 40) | 15 (± 14) | 15 (± 27) | 0.27 |

**Table 20. Provider experience characteristics according to questionnaire response to “Additional training on the emotional / psychological aspects of MAID would be beneficial for me”. Analysis completed using Kruskal-Wallis test.**

|  | **1** | **2** | **3** | **4** | **Total** | **P-value** |
| --- | --- | --- | --- | --- | --- | --- |
|  | **(N=13)** | **(N=19)** | **(N=4)** | **(N=2)** | **(N=38)** |  |
| **Years as healthcare provider** |  |  |  |  |  |  |
| Median (IQR) | 28 (± 14) | 19 (± 15) | 20 (± 9.0) | 27 (± 1.5) | 22 (± 16) | 0.68 |
| **Years as MAiD provider** |  |  |  |  |  |  |
| Median (IQR) | 7.0 (± 2.0) | 6.0 (± 3.5) | 8.0 (± 1.5) | 5.0 (± 2.0) | 7.0 (± 3.0) | 0.60 |
| **Number of MAiD provisions in last 12 months** |  |  |  |  |  |  |
| Median (IQR) | 12 (± 20) | 12 (± 20) | 9.0 (± 13) | 28 (± 8.0) | 12 (± 22) | 0.52 |
| **Number of hours spent on MAiD work per month** |  |  |  |  |  |  |
| Median (IQR) | 25 (± 25) | 10 (± 15) | 8.0 (± 4.5) | 25 (± 5.0) | 15 (± 27) | 0.17 |

**Table 21. Provider experience characteristics according to questionnaire response to “Ideally, I would complete fewer cases of MAID”. Analysis completed using Kruskal-Wallis test.**

|  | **1** | **2** | **3** | **4** | **5** | **Total** | **P-value** |
| --- | --- | --- | --- | --- | --- | --- | --- |
|  | **(N=1)** | **(N=5)** | **(N=4)** | **(N=20)** | **(N=8)** | **(N=38)** |  |
| **Years as healthcare provider** |  |  |  |  |  |  |  |
| Median (IQR) | 16 (± 0) | 19 (± 14) | 21 (± 14) | 22 (± 17) | 29 (± 11) | 22 (± 16) | 0.68 |
| **Years as MAiD provider** |  |  |  |  |  |  |  |
| Median (IQR) | 5.0 (± 0) | 6.0 (± 2.0) | 6.5 (± 1.5) | 7.0 (± 4.3) | 6.5 (± 2.5) | 7.0 (± 3.0) | 0.78 |
| **Number of MAiD provisions in last 12 months** |  |  |  |  |  |  |  |
| Median (IQR) | 6.0 (± 0) | 30 (± 20) | 33 (± 67) | 12 (± 16) | 10 (± 7.0) | 12 (± 22) | 0.37 |
| **Number of hours spent on MAiD work per month** |  |  |  |  |  |  |  |
| Median (IQR) | 6.0 (± 0) | 40 (± 52) | 23 (± 48) | 14 (± 13) | 14 (± 30) | 15 (± 27) | 0.77 |

**Table 22. Provider experience characteristics according to questionnaire response to “I feel my current workload relating to MAID is sustainable in terms of its impact on my wellbeing over time”. Analysis completed using Kruskal-Wallis test.**

|  | **1** | **2** | **3** | **4** | **5** | **Total** | **P-value** |
| --- | --- | --- | --- | --- | --- | --- | --- |
|  | **(N=5)** | **(N=11)** | **(N=9)** | **(N=11)** | **(N=2)** | **(N=38)** |  |
| **Years as healthcare provider** |  |  |  |  |  |  |  |
| Median (IQR) | 28 (± 9.0) | 21 (± 14) | 19 (± 9.0) | 28 (± 15) | 11 (± 1.5) | 22 (± 16) | 0.37 |
| **Years as MAiD provider** |  |  |  |  |  |  |  |
| Median (IQR) | 6.0 (± 2.0) | 5.0 (± 4.5) | 7.0 (± 1.0) | 7.0 (± 2.5) | 5.0 (± 1.0) | 7.0 (± 3.0) | 0.52 |
| **Number of MAiD provisions in last 12 months** |  |  |  |  |  |  |  |
| Median (IQR) | 6.0 (± 7.0) | 10 (± 14) | 15 (± 10) | 25 (± 18) | 6.0 (± 4.0) | 12 (± 22) | 0.05 |
| **Number of hours spent on MAiD work per month** |  |  |  |  |  |  |  |
| Median (IQR) | 4.0 (± 15) | 9.0 (± 20) | 11 (± 28) | 20 (± 30) | 11 (± 9.0) | 15 (± 27) | 0.24 |

**Table 23. Provider experience characteristics according to questionnaire response to “I am aware of other healthcare workers who have removed themselves from their work with MAID due to burnout (or other reasons relating to emotional / psychological distress)”. Analysis completed using Kruskal-Wallis test.**
